# Supplementary material for: Are carbon emissions trading and green financial instruments synergistic? -Comprehensive quantitative research based on content analysis
Source: PLoS One. 2024 Mar 7;19(3):e0298601. doi: 10.1371/journal.pone.0298601 (PMC10919601; doi:10.1371/journal.pone.0298601)
Supplement: S1 Data — (ZIP) [file pone.0298601.s001.zip › Data/地级市Z_F工作报告.zip!/╣π╢1⁄2/╔╟╬▓╩╨/└┤╫╘╣1⁄2╓┌║┼í╛┬φ┐╦╩2╛▌═°í┐_2018.docx]

各位代表：

我代表市人民政府向大会作政府工作报告，请予审议，并请政协各位委员和其他列席人员提出意见。

2017年工作回顾

过去一年，在以习近平同志为核心的党中央坚强领导下，按照省委、省政府的部署，根据市委的安排，市政府坚持以习近平新时代中国特色社会主义思想为指导，认真学习贯彻党的十八大、十九大精神，全面落实习近平总书记对广东工作的重要指示批示精神，牢固树立新发展理念，统筹做好稳增长、促改革、调结构、惠民生、防风险各项工作，经济社会呈现持续健康发展的良好态势。

（一）经济发展水平明显提升。坚持超前研判、精准施策、链式推进，主要经济指标增幅位居全省前列。全市地区生产总值（含深汕特别合作区）903亿元，增长8.1%；固定资产投资754.4亿元，增长17%；规模以上工业增加值284.1亿元，增长11.4%，增速排名全省第一；工业用电量23.5亿千瓦时，增长10.1%，增速排名全省第三；一般公共预算收入36.8亿元，增长23.9%，增速排名全省第一；一般公共预算支出222.6亿元，增长7.7%；城镇和农村常住居民人均可支配收入分别为24086元、13501元，增长7.6%和8.5%。始终坚持扩大有效投资，完成工业投资299.3亿元，增长45%，增速排名全省第一，工业投资、基础设施投资、房地产投资占固定资产投资比重分别为40%、28%和13%。制定出台一系列实体经济扶持政策，新培育“四上”企业97家，新投入工业技改资金100亿元。完成社会消费品零售总额593亿元，增长8%，居民消费价格指数上涨0.7%。实际利用外资1亿美元，增长125.2%。深入实施创新驱动发展战略，新增高新技术企业9家、企业研发中心36家，新认定省级工程技术研究中心5家，新增科技企业孵化器、众创空间面积5.8万平方米，高技术制造业增加值占规模以上工业增加值比重提高到20.4%，专利申请量和授权量分别增长70%和17%，高新技术产品出口占汕头关区总量的47%。我市主导制定的全省首个镀金属饰品行业标准正式实施。电子信息、电力能源、新能源汽车等主导产业不断壮大，一批层次高、支撑力强的骨干企业发挥效益。全年接待过夜游客845万人次，旅游总收入130亿元，分别增长7%和6.9%，承办“5·19”中国旅游日广东省主会场活动，新增2家国家4A级景区，红海湾成功入选省首批特色小镇和“互联网+旅游”小镇，陆河华侨城·罗溪谷预计今年初正式开放运营。实现农业增加值141.8亿元，增长4.8%，省、市级农业产业化龙头企业达77家，新增省级农业标准化示范区4个。实现海洋经济总产值358亿元，增长10%。

（二）产业共建工作全面推进。深入实施向西融珠战略，全面参与深莞惠+汕尾河源“3+2”经济圈建设，积极推进产业共建，“总部研发销售在深圳，生产基地在汕尾”的产业共建模式更趋成熟。突出抓好“1+4”产业园区载体建设，投入园区开发建设资金25亿元。深化园区体制机制改革，授予汕尾高新区67项市级管理权限，实行“一站式”审批、“封闭式”管理。全面开展新一轮大招商行动，推动产业项目加快落地建设，全市“1+4”产业园区全年建成投产项目11个，新落地动工项目56个（其中投资亿元以上项目35个），已供地准备动工项目28个，比亚迪新能源汽车、信利TFT5代线、国信通科技、海王医药、香雪制药、华为云服务等一批产值超十亿、超百亿产业项目相继开工建设、试产投产。省委、省政府在我市召开粤东西北协调发展暨产业共建工作现场会，对我市产业共建工作给予充分肯定。

（三）城市功能品质显著增强。铁公机港等交通基础设施建设力度进一步加大，全年投入资金28.6亿元。铁路建设取得重大突破，深汕城际捷运开通运营，广汕高铁汕尾先行段动工建设，汕汕高铁前期工作加快推进，启动深汕高铁、龙汕铁路前期工作。高快速路网建设全面铺开，新改建公路里程143公里，兴汕高速加快建设。深汕西高速改扩建、珠东快速、广东滨海公路汕尾段前期工作加快推进。市政路网不断完善，汕尾大道完成改造升级，红海西路建成通车，东城路加快改造建设，汕马路、通航路、红海中路、红海东路、香洲路、康平路等一批市政道路全面拓宽优化。国道324陆丰穿城段改建工程、海丰莲花大道、陆河大道南段、汕遮路、华侨南三线公路等县区重要干道加快建设。汕尾机场启动前期工作。小漠国际物流港、甲湖湾电厂10万吨级煤码头、陆丰（粤东）核电厂重件码头工程建设加快推进。中心城区扩容提质扎实推进，汕尾市城市总体规划正式获批，市区建成区面积30平方公里，较两年前的16.89平方公里拓展了80%。加大土地收储出让力度，全市完成土地征收5.03万亩（含协议征地），其中已入库2.13万亩，市区完成征地1.73万亩，全市土地出让收入107.5亿元，是过去5年总和的1.65倍，中央商务区6宗地块拍出高价，表明外界很看好汕尾的发展。城市功能不断增强，市民体育中心、善美广场建成开放，环品清湖滨海景观带提升改造工程进入招投标程序，市区新建公共停车场10处、新开通公交线路7条、建成WLAN303个。新区三个起步区建设全面提速，完成固定资产投资50.8亿元。中央商务区功能日趋完善，站前广场、站前路及周边配套设施建成投入使用，保利大都汇、农商行大厦、万升广场、深汕中心医院、市民服务广场及碧桂园、星河湾、恒大、雅居乐等相继进驻，一批城市综合体及星级酒店动工建设。开展声势浩大的“创文”“创卫”活动，全面推进文明城市、文明县城、文明镇街、文明村居“四级联创”，坚持从最薄弱的环节抓起，从公众最需要的地方入手，集中开展卫生环境、交通秩序、违法建设和户外广告综合整治专项行动，向城乡环境“脏乱差堵”宣战，全面清理打击乱搭乱建、乱摆乱卖、乱堆乱放“六乱”行为，升级改造农贸市场81个，拆除各类违建72万平方米，其中市区拆除18万平方米。深入开展地毯式安全生产隐患排查整治工作，共排查23.7万家企业及场所，发现事故隐患7.7万处，完成整改6.7万处。开展“喜看汕尾新变化”等创文系列活动，新增注册志愿者2.19万人。成功创建成为省卫生城市，获省文明城市提名城市称号，省同意我市提前一年在2018年创建省文明城市。

（四）改革开放活力有效释放。全面完成市、县两级行政审批标准化改革，取消调整90项行政审批事项，清理规范第二批29项行政审批中介服务事项。实施新一轮“放管服”改革，社会投资、政府投资项目审批时间分别缩短为40天、55天，平均提速70%以上。“一门式一网式”改革成效明显，市政务服务中心进驻事项734项，行政审批事项网上全流程办理率93.2%，高效便利的办事流程获《人民日报》报道点赞。新增各类市场主体2.8万户，注册资本（金）211.9亿元，分别增长122%和42%。加快推进供给侧结构性改革，去房地产库存7万平方米，清理“僵尸企业”22户，降低企业成本6.5亿元，投入23.8亿元补齐农村电网、燃气管网等基础设施短板。加快投融资体制改革，搭建交通投融资平台，出资5000万元与广东粤科金融集团合作成立创业投资基金，成功发行市级第一支企业债，募资11亿元。实施新一轮城市执法体制改革，完成食盐、公路、住房公积金及代建局等管理体制改革，精简优化红海湾开发区、华侨管理区机构编制。推动农村集体产权制度改革。扎实推进农村综合水价改革。完成普惠金融“村村通”试点工作。陆河县水唇镇被列入“全国农村综合改革试点单位”。对外开放继续深化，积极参与国家“一带一路”建设，加强闽粤赣十三市交流合作。实现全市口岸通关一体化全覆盖，完成进出口总额199亿元。

（五）生态环境质量持续改善。坚决落实中央环保督察问题整改，坚守生态红线和环保底线，大力推进绿色低碳循环发展。落实节能减排责任，超额完成电机能效提升和绿色建筑发展任务，关停非法电镀厂28家。大力发展绿色交通，淘汰黄标车1796辆，新投放纯电动新能源公交车600辆，全市保有量达1050辆，占公交车总数86.4%。实施大气污染防治行动计划，主要污染物减排实现年度目标，AQI达标率、PM2.5、PM10、NO2、O3等指标连续三年均稳居全省第一。落实水污染防治工作计划，加强饮用水源管理保护，城市集中饮用水源、国考省考断面、主要入海河口、近岸海域环境功能区水质达标率均为100%，赤沙水厂动工建设。全面推行河（库）长制，全市784条河流、392宗水库均落实专人负责。品清湖蓝色海湾整治及奎山河、奎山湖黑臭水体整治启动实施，龟龄岛保护与开发利用项目有序推进。华润海丰电厂被列为省近零碳排放试点企业。陆河、海丰生活污水处理PPP项目进展顺利，市生活垃圾无害化处理中心二期、陆丰东南生活垃圾焚烧发电厂、市医疗废物无害化集中处理中心动工建设。“一县一场、一镇一站、一村一点”收运处理体系高效运作，建成58个镇（街）转运站和4615个村收集点，农村生活垃圾有效处理率达92.7%，村庄保洁覆盖率100%。全面开展禁养区整顿清理，关停和搬迁畜禽养殖场户370个。推进新一轮绿化大行动，人工造林14.4万亩，建设生态景观林带69.6公里，实施山体复绿24.8万平方米。

（六）社会民生事业全面发展。始终践行以人民为中心的发展思想。获得国家、省专项扶持资金64亿元，争取省的支持，获批政府债券35.2亿元。全年完成九大民生支出174.5亿元，占一般公共预算支出78.4%。市下拨6.4亿元用于支持基层建设，每个镇（街）综合保障专项经费100万元，每个镇（街）消防安全整治专项经费100万元（11个重点整治镇各300万元），142个省定贫困村创建新农村示范村专项经费各100万元，每个村（社区）环境卫生整治专项经费5万元，提高村级办公经费至每年7万元，提高村干部补贴标准至每人每月2400元，陆丰三甲地区每个禁毒重点村专项经费各50万元，给予获国家级或省级的文明镇（街）、文明村（社区）、文明单位20万至80万元奖励，给予每月创文测评先进的镇（街）、村（社区）和市直单位5000至5万元奖励。城镇新增就业4.9万人，新增转移农村劳动力就业2.8万人，城镇登记失业率2.38%，基本社会保险覆盖率96%。拓展提升“人才驿站”，出台“红海扬帆人才计划”，设立2亿元人才基金招贤纳士。教育“创现”工作全面启动，新增幼儿园27所，清理整治无证幼儿园112所，成功创建2所省重点中职学校，总投资8.75亿元、在校生规模达10000人的市高级技工学校破土动工，市、县两级教师发展中心建设进展顺利，新增名校长、名教师、名班主任工作室25个，引进研究生学历教师38名，成功引进市林伟华中学和彭湃中学两位名校长，名师名校长引才计划取得突破。所有改革医院全部取消药品加成，医联体试点建设扎实推进，中山大学深汕中心医院动工建设，市妇女儿童医院、第三人民医院建设进展顺利，甲子、碣石、梅陇3家综合性医院建设稳步推进，21家标准化乡镇卫生院和175家规范化村卫生站建设基本完成，每万常住人口全科医生数增长51%。文化、体育等社会事业蓬勃发展，举办群众音乐舞蹈花会等一系列文体盛会，西秦戏《马援伏波》获省第十三届艺术节剧目二等奖，皮影戏《哪吒闹海》获第二届南充国际木偶艺术周优秀传承奖，成功举办市第一届运动会，6家单位、3名个人获得全国群众体育先进单位及先进个人称号，市体校和业余体校新校区完成立项选址征地工作。精准扶贫、精准脱贫基础不断夯实，累计投入12.6亿元，实施扶贫项目1247个，转移就业1.5万人，实现5.158万相对贫困人口脱贫，率先实现所有行政村100%光纤到户。社会矛盾排查化解有效加强，民间矛盾纠纷调解成功率达99.8%，社会和谐稳定持续向好。食品药品行业连续多年未发生重大安全事故，陆丰市获评省首批国家农产品质量安全县（市）。省、市十件民生实事扎实推进。

（七）政府自身建设不断加强。自觉接受人大、政协监督，坚持向市人大及其常委会报告工作，向市政协通报情况，共办理市人大代表建议50件、市政协提案120件，市政府负责人牵头办理人大重点建议7件，《汕尾市品清湖环境保护条例》等地方性法规获准实施。深入推进政务公开和各领域办事公开，自觉接受社会监督和舆论监督，坚持倾听民声、收集民情，12345投诉举报平台接通率位列全国第一方阵。推行政府法律顾问和市政府负责人出庭应诉制度，行政复议和行政应诉工作不断加强，行政行为逐步规范。推进“两学一做”学习教育常态化制度化，严格落实中央八项规定精神，驰而不息纠正“四风”，“三公”经费下降9%。持续推进政务整治、正风肃纪集中行动，从严查处“为官不为”问题299件，处理577人。全面加强审计工作，推行重大项目和重点工作限时办结制，实施全覆盖、常态化的绩效考核和督查问责，政府公信力、执行力明显提升，政务环境全面好转。

（八）深圳对口帮扶力度持续加大。深圳全面实施新一轮对口帮扶汕尾工作，以前所未有的力度，从产业、城建、交通、改革、民生各个领域全方位帮扶汕尾，投入帮扶资金11.5亿元，帮助争取园区贷款贴息资金3217万元。给予落户“1+4”共建园区的企业享受深圳各区同等政策待遇，推动重大产业项目把生产制造环节落户汕尾，帮扶引进产业项目62个，其中由深圳4个区结对帮扶引进的比亚迪、海王医药、国信通科技、威翔航空等一批重大产业项目发挥了龙头带动作用。深圳各帮扶单位全力推动精准扶贫精准脱贫工作，结对帮扶汕尾109个村，实施帮扶项目575个，推动驻村集体收入和贫困户人均纯收入分别增长154%、50%。投入16亿元援建中山大学深汕中心医院，全资援建市和海丰县教师发展中心、市城区盐町头小学等一批民生项目。深圳市4个区、38个市直部门、235名新一轮驻汕帮扶干部，真心真情帮扶汕尾，不仅给汕尾带来了实实在在的项目和资金，更带来了深圳的国际视野、开放思维、先进理念和高效作风。

此外，外事侨务、国防建设、港澳和对台工作有新进步，民族宗教、人事编制、民政、统计、物价、供电、供销、打假、工会、青年、妇女儿童、残联、红十字会、老促、人防、档案、方志等工作取得新成绩。

各位代表，过去一年，我们牢固树立争先意识、质量意识、实干意识、担当意识，激发广大干部群众大打翻身仗的血性和斗志，攻克了许多长期想解决而没有解决的难题，办成了许多事关全局影响长远的要事，办妥了许多百姓期许而没有办成的实事，办好了许多干群称道的喜事。我们敢于打破常规，以踏石留印、抓铁有痕的劲头，大干快上，刷新一项又一项纪录。继红海西路拆迁到建成通车仅用1年、陆河比亚迪项目签约到投产仅用4个月后，深汕中心医院征地、迁坟、清表仅用40天，市高级技工学校征地、迁坟、清表仅用27天。我们敢于奋勇争先，以不甘居后、拼搏进取的精神状态，推动多项工作“提档进位”。GDP、规模以上工业增加值等主要经济指标增幅走在全省前列；产业共建走在全省前列，亿元以上新动工项目35个，大幅超额完成省定任务，全省排名第一；新农村建设走在全省前列，率先完成省定贫困村创建新农村示范村整治规划编制，“三清理、三拆除、三整治”工作受到省通报表扬；“智慧人社”建设走在全省前列；政府公共服务总体满意度跃升至全省第14名，居粤东西北地区第5名，开办企业便利度跃升至全省第11名，环保责任制考核、粮食安全责任考核均跃升至全省第14名，气象现代化考核从2012年全省末位跃升至第15名，社会治安综合治理考核获省肯定。我们敢于攻坚克难，以壮士断腕、剔肤见骨的胆魄和决心，以逢山开路、遇水搭桥的勇气和智慧，坚决扫除长期制约汕尾发展的沉疴积弊。在国家和省禁毒委特别是省公安厅的大力指导支持下，举全市之力铁腕禁毒，市政法、公安等市直部门和陆丰市下大决心开展艰苦卓绝的奋战，全面完成了国家摘除“毒帽”的6条标准、100项任务，去年3月份以来陆丰未发现制毒窝点，两年多来抓获制贩毒逃犯达514人，彻底扭转陆丰毒品泛滥的严峻形势，得到国家禁毒委、省禁毒委的充分肯定，同意陆丰市向国家禁毒委申报“摘帽”，从上世纪90年代中期出现、严重困扰汕尾20多年的制贩毒问题可望成为历史。在国家全面禁止“洋垃圾”入境之际，全市上下以没有退路的决心，采取“清门店、拆栅寮、堵货源、抓为首”等强有力措施，全力开展打击整治碣石地区走私经营“洋垃圾”专项行动，先后清理门店3279间，拆除栅寮3017间，收缴旧服装9060吨，彻底切断海陆进出渠道，惩处一批违法分子，绝大多数从业人员得到妥善转产转业，38年来久禁不止的碣石“洋垃圾”问题得到根本性遏制，大规模走私经营“洋垃圾”成为历史。我们直面城市管理的顽瘴痼疾，热切回应人大代表、政协委员和广大市民对取缔非法载客三轮车的强烈呼声，充分考虑大部分三轮车工友属困难群体的实际，投入三轮车回收补贴资金790万元，提供4052个企业就业岗位、388个政府公益岗位，推出低保、临时困难救助等一系列扶持政策。市城管、公安、残联、人社、民政、交通、宣传、工商、团委等部门和市城区、红海湾开发区的区、镇（街）、村（居）三级全体动员、全力以赴，对三轮车工友实行“六包”，深入细致倾听解决每位工友的诉求和困难，短短25天全部回收载客三轮车3081辆，从上世纪90年代中期出现的市区和红海湾载客三轮车已成为历史，为下一步持续整治交通秩序创造了良好条件，也为我们今后扎实深入做好群众工作、解决城市发展中的老大难问题提供了范例。在这次取缔工作中，广大三轮车工友顾全大局、纯朴善良，积极主动支持配合政府的取缔行动，是可敬可爱的好市民，我们一定要善待这些工友，继续解决好他们的工作、生活困难，并切实优化完善市区公共交通，满足市民出行需求。实践证明，只要各级党政高度重视、领导干部率先垂范、相关部门齐心协力、广大群众支持配合，上下同欲，铁定决心，真抓实干，就没有迈不过的坎、闯不过的关，就没有干不成的事、解决不了的难题。

过去一年取得的成绩，是省委、省政府和市委正确领导的结果，是全市360多万干部群众勤劳智慧的结晶。在此，我谨代表汕尾市人民政府，向广大工人、农民、知识分子、干部职工，向驻汕人民解放军、武警官兵和各民主党派、人民团体、外出乡贤、社会各界人士，向长期关心支持我市经济社会发展的港澳同胞、台湾同胞、海外侨胞和国际友人，致以崇高的敬意和诚挚的感谢！

过去一年我市经济社会虽然取得了长足进步，但对照党的十九大报告提出的要求，我市经济社会发展中仍然存在许多不平衡不充分的矛盾和问题。一是经济发展不充分。经济水平与珠三角等发达地区有较大差距，GDP、财政收入分别仅为全省的1%、0.3%，人均GDP仅为全省的1/3。主导产业不突出，产业聚集度不高，创新能力弱，实体经济水平低，全市“四上”企业仅有584家，没有境内上市企业，国家高新技术企业仅有21家，R&D占GDP比重仅为0.85%。二是“三农”发展水平较低。村庄规划滞后，农房建设无序，有新房无新村现象突出，村居环境脏乱差现象普遍。农村基础设施、公共设施欠账较多。农业生产规模化、产业化水平不高，农民收入水平偏低。三是城市规划建设管理水平不高。规划编制的科学性不强、衔接性不够、执行刚性约束不足。中心城区首位度仍需加强，没有市级博物馆、档案馆、文化馆、纪念馆、工人文化宫、少年宫，城市公共服务配套设施亟待完善。四是污染防治任务艰巨。部分地区水污染问题较为突出，城市黑臭水体整治还不彻底，近海养殖造成的污染严重，污水处理设施配套管网建设滞后，个别地方土壤污染问题比较突出。五是民生事业短板突出。精准扶贫任务重，还有3.7万人口尚待脱贫。普惠性学前教育不足，义务教育城乡差距较大，职业教育发展滞后，没有本科高校，教育质量整体不高，高考本科率、重本率长期排在全省末位。基层医疗卫生服务能力还有待提升，每千常住人口床位数、每万常住人口全科医生数和县域内住院率远低于全省水平。六是公共安全面临诸多挑战。社会治安形势依然严峻复杂，维护社会稳定压力大，陆丰禁毒“摘帽”成果巩固工作任务艰巨。安全生产形势不容乐观，高危行业和易发事故的领域多、潜在风险大，公平“12·9”火灾事故充分暴露出消防安全生产还存在许多薄弱环节，给我们敲响了警钟。七是政府自身建设有待加强。行政审批和管理事项仍然较多，部分行政机构设置还不够合理，政府办事流程不优、效率不高，一些干部法治意识、创新意识、担当意识、实干意识和纪律观念不强，能力不足、本领不够，存在不作为、慢作为甚至乱作为的现象。基层治理能力较为薄弱。对这些问题和挑战，我们必须高度重视，切实加以解决。

2018年工作安排

2018年是贯彻党的十九大精神的开局之年，是改革开放40周年、汕尾建市30周年，是决胜全面建成小康社会、实施“十三五”规划承上启下的关键一年。做好今年政府工作，必须聚焦落实党的十九大提出的新目标新要求新思路新举措，清醒认识和深刻把握市情实际，找准新时代汕尾发展的立足点和发力点，以更高的站位、更广的视野和更大的作为，一步一个脚印，踏踏实实干好工作，为把广东建设成为向世界展示习近平新时代中国特色社会主义思想的重要“窗口”和“示范区”作出贡献。

中央经济工作会议明确提出了习近平新时代中国特色社会主义经济思想，深刻阐述了“七个坚持”的主要内涵，这是中国特色社会主义政治经济学的最新成果，是我们做好当前和今后一个时期经济工作的根本遵循。我们必须坚决贯彻落实习近平新时代中国特色社会主义经济思想，把思想和行动统一到以习近平同志为核心的党中央关于经济工作的重大判断、重大决策和思想方法上来，按照省委、省政府的统一部署，牢牢把握高质量发展的根本要求，转换发展动力，着力解决发展不平衡不充分问题，努力实现更高质量、更高效率、更加公平、更可持续的发展。

高质量发展是满足人民日益增长的美好生活需要的发展，是保持经济持续健康发展的必然要求。从供给看，就是要发展先进产业，把互联网、智能制造、创新融入到制造业等产业中。从需求看，就是要满足个性化、多样化的需要，改变过去仅提供温饱型的需求供给，通过需求来刺激供给，制造更多适销对路的产品。从投入产出看，要提升劳动效率、资金效率、土地效率、资源效率、环境效率，着力提高科技贡献率和全要素生产率。从分配看，要坚持投资有回报、企业有利润、员工有收入、政府有税收，避免出现亏本买卖、重复建设、低端落后等难以为继的发展。高质量发展是我们必须树立的重要发展理念。当前，我们的发展存在经济总量小和质量效益差的双重压力，三次产业结构为15.7:44.5:39.8，第三产业比重低，现代服务业增加值占服务业比重、生产性服务业增加值占服务业比重分别为40.9%、33.9%，远低于全省的61.7%、52.5%，高技术制造业增加值占规模以上工业增加值比重为20.4%，低于全省的27.1%，单位面积土地GDP产值、一般公共预算收入分别为每平方公里1700万元、63万元，远低于全省的4400万元、578万元。我们要切实增强推动高质量发展的自觉性和坚定性，牢固树立符合高质量发展要求的正确政绩观，不走短期刺激、低端发展的旧路，不走先污染后治理的错路，不能唯量而量、唯速度而速度，宁可降点速度，也要更好、更有效、更高端的发展，通过质的提升，进而达到量的扩张，通过质量、效益、动力转换，实现速度、规模、总量的提升。

如何实现高质量发展，对汕尾来说，最重要的是建设现代化经济体系，要牢牢把握质量第一、效益优先的要求，牢牢把握供给侧结构性改革的工作主线，牢牢把握质量变革、效益变革、动力变革的基本路径，牢牢把握加快建设协同发展的产业体系这一着力点，牢牢把握构建社会主义市场经济体制这一制度保障。具体来说，要重点把握以下几个方面：一要建立先进高端的现代化产业体系。要通过产业共建推动珠三角优势产业、优质企业把生产环节布局我市，引进提升研发、销售环节，大力发展先进制造业和新兴产业。要推动物流业、金融业与制造业联动发展，立足资源禀赋和区位优势打造珠三角旅游健身养老基地，以生产性服务业为重点发展壮大现代服务业。要突出抓好深外海捕捞和水产品深加工，加快退出近海养殖，提升海洋渔业发展水平。要通过集中丢荒农田发展种养业，加快农业适度规模经营，提高农业产业规模效益。二要节约集约利用土地。要实行更加严格的土地管理制度，从严控制园区建设用地供给，严格执行入园项目投入、产出、用地、容积率等刚性约束标准。要通过城市更新等方式有序推进存量建设用地优化利用，从“土地存量”找出“发展增量”。三要坚守环保底线。要树立绿色发展理念，宁愿发展慢一点，也要好一点，决不能饥不择食、急功近利。要加大生态环境治理和保护力度，着重补齐污水垃圾处理设施等短板。四要加强人才引进和培养。要树立人才是第一资源的理念，厚植高质量发展的根基。针对产业发展的现实需求，进一步拓宽人才引进渠道，优化人才环境，推动“人才驿站”和“红海扬帆人才计划”等政策措施落地见效，确保人才引进取得实质性进展。五要防范和控制风险。要树立“输不起”意识，处理好稳与进的关系，既不能为了稳而止步不前，更不能因风险失控造成得不偿失的破产经济。目前汕尾的综合负债率处于全省较低水平，要在严格管控风险的基础上，不局限于“有多少钱办多少事”思维定势，实施更加积极的财政政策，继续撑大有效投资的盘子，实现有效的高质量发展。六要营造良好营商环境。要敢于通过改革清除制约生产效率和资源配置效率提高的体制机制障碍，加快推进“放管服”改革，继续推出减负降成本措施，制订鼓励先进产能、动力转换、结构调整的激励引导政策。要坚决改变“要么勾肩搭背，要么背对背”的畸形政商关系，畅通政府企业沟通渠道，“打开前门”主动服务，“摆上台面”积极帮助，在公平法治的前提下正大光明地为企业和企业家服务，积极构建亲清新型政商关系。

2018年政府工作的总体要求是：以习近平新时代中国特色社会主义思想为指导，全面贯彻党的十九大精神，坚决维护以习近平同志为核心的党中央权威和集中统一领导，按照“三个定位、两个率先”和“四个坚持、三个支撑、两个走在前列”的要求，落实省委十二届二次、三次全会和市委七届四次、五次全会的部署，坚持稳中求进工作总基调，坚持新发展理念，紧扣我国社会主要矛盾变化，贯彻高质量发展的要求，统筹推进“五位一体”总体布局和协调推进“四个全面”战略布局，以供给侧结构性改革为主线，深入实施创新驱动发展战略，大力推进改革开放，坚定向西融珠发展，积极融入粤港澳大湾区，全面推进深汕产业共建，在打好防范化解重大风险、精准脱贫、污染防治的攻坚战方面取得扎实进展，加强和改善民生，把汕尾建设成为宜居宜业宜游现代化滨海城市，为全面建成小康社会、开启全面建设社会主义现代化新征程打下坚实基础。

今年经济社会发展的主要预期目标是：地区生产总值增长8%；人均生产总值增长7%；规模以上工业增加值增长11%；固定资产投资增长16%；社会消费品零售总额增长10%；外贸进出口总额增长3%；一般公共预算收入增长10%；城乡居民人均可支配收入增长8.2%；居民消费价格涨幅控制在3%左右；城镇登记失业率控制在3%以内；节能减排约束性指标完成省下达年度任务。

实现今年的目标任务，重点是抓好以下九个方面工作：

一、推进供给侧结构性改革，加快构建现代化经济体系

——加快发展制造业。落实《广东省沿海经济带综合发展规划》，加大招商引资力度，充分利用现有产业基础和临海资源禀赋，引进培育和发展壮大先进制造业，力争引进年产值5000万元以上产业项目100个，先进制造业增加值占规模以上工业增加值比重达40%以上，打造承接珠三角辐射粤东的战略支点和战略平台。以汕尾高新区为主阵地，发挥信利、信元、国信通等龙头企业带动作用，打造电子信息产业基地。以新能源汽车产业为依托，加快红草比亚迪汽车零部件新材料项目投产、陆河比亚迪新能源汽车达产，促进上下游企业配套集聚，打造新能源汽车产业基地。依托海王、香雪、江涛等龙头企业，培育壮大生物医药新兴产业。推动在陆丰设立电力、海工装备制造基地。加快公平、可塘、梅陇、三甲等环保集聚区建设，扶持纺织服装、金银首饰、健康食品、海产品等传统优势产业发展，推动传统产业转型升级。支持鼓励企业利用先进设备和适用技术改造提升，大力推广应用“机器换人”。实施质量强市战略，鼓励企业争创名牌和参与行业标准制修订。

——着力壮大现代服务业。大力发展旅游休闲、现代物流、信息技术、现代金融等现代服务业。推进全域旅游发展，加强旅游策划和推广，加快红海湾、陆河全域旅游示范区建设，推进红海湾“互联网+旅游”小镇建设，大力发展滨海及乡村旅游。加快现代旅游标准化建设，推动陆河温泉之乡、华侨城·罗溪谷、螺溪白水寨、陆丰金厢周恩来渡海处、华侨四季果园、海丰黄羌林场运动小镇等景区景点规划开发建设，启动中国农运摇篮红色经典景区策划工作，打造全省乃至全国著名滨海旅游休闲目的地。加快华侨古建筑材料市场、市区水产品交易市场等一批物流项目建设。发挥好消费的基础性作用，挖掘汽车、家电等传统消费潜力，支持城镇居民改善性住房需求和新市民住房消费，大力发展旅游休闲、健康养老、在线教育、文化创意、体育健身等服务消费，打造万达广场、信利中央广场、万升广场、保利大都汇、保利金町湾、农商行大厦、星河湾商业、碧桂园商业、大中华商业、增达商业等一批高端城市综合体。深入实施电子商务、快递进社区进农村工程，推动实体店销售和网购融合发展。

——加快推进产业共建。借力深圳全面对口帮扶，推动深圳先进产业生产环节、生产基地布局汕尾，打造珠江东岸产业转移主承接区，力争承接珠三角和深圳转移产业项目50个以上。加快产业园区项目建设，力争园区实现新动工工业项目50个，建成投产工业项目37个，实现工业产值600亿元以上。以深汕特别合作区体制机制调整为契机，积极向省争取对我市省级产业园调整升级“一揽子”政策，推动红草产业园区升级调整为深圳市对口帮扶的省级产业园区。加快园区扩园步伐，加快红草园区向周边扩容，支持海丰加快“一城三园”建设，支持陆丰发展“一园多片区”，支持陆河规划建设第二产业园区。投入24亿元加快园区路水电气讯及污水处理等基础设施建设，完善金融、物流、检验检测等公共服务配套功能。

——大力降低实体经济成本。深入推进“三去一降一补”，在降低企业制度性交易成本、税费成本、要素成本上协同发力，落实好国家“降税减证提标”三项措施和省“实体经济十条”。抓住省出台《海陆丰革命老区振兴发展规划》有利契机，加强与省相关部门沟通对接，加大向上争取政策资金工作力度，用好用活用足海陆丰革命老区扶持政策，争取在财政、产业、金融、人才、投资政策、土地政策等方面的扶持上有新突破。用好省产业共建普惠性和叠加性政策奖补措施，进一步优化土地使用、招商引资、人才引进、技术创新等优惠政策。进一步摸清企业实际困难，研究出台新的降本举措。清理摊派事项和各类达标评比活动，最大程度减轻企业负担。

二、实施创新驱动发展战略，持续激发创新发展活力

——加快创新科技载体建设。把“1+4”产业园区作为创新驱动发展的主战场，推动深汕特别合作区与汕尾高新区纳入深圳国家自主创新示范区拓展区、汕尾高新区申报国家级高新区，加快海丰生态科技城、陆丰产业转移园、陆河新河工业园申报省级高新区。加快创建省特色小镇，开展专业镇协同创新中心建设，坚持“一镇一主业”发展模式，做大做强创新型产业集群。

——大力提升自主创新能力。积极参与广东省“国家科技产业创新中心”建设，组织推荐重大科技专项和应用型研发专项，重点支持电力能源、电子信息、现代农业、新材料、新能源汽车、生物医药等领域关键环节技术攻关。加强高新技术企业扶持和培育，力争新增国家高新技术企业15家。支持企业与高校、科研院所共建研发机构，新增各类研发机构、研究中心、实验室等30家，规模以上工业企业设立研发机构比例提高到40%，力争主营业务收入5亿元以上工业企业实现研发机构全覆盖。推广“信利创新”经验，鼓励扶持企业加快科技创新及成果转化，争取专利申请量突破2500件、授权量突破1000件，PCT专利申请10件以上。加快科技企业孵化器、加速器多元化建设，实现产业园区科技企业孵化器全覆盖。

——加强创新人才队伍建设。坚持人才优先发展，大力实施“红海扬帆人才计划”，支持创新型企业聚集、引进、培育高层次领军型创新人才，对引进的两院院士、国家科学技术奖获得者，国家重点学科、重点实验室、工程研究中心、工程实验室的主要专家，省级有突出贡献的中青年专家、重点学科带头人等领军人才，财政分别一次性给予500、200、100万元专项工作经费和100、80、50万元住房补贴。落实人才培养与激励保障的政策措施，切实解决新引进的高层次人才住房、入户及其配偶就业、子女入学入托等问题。

三、全力扩大有效投资，加快现代化基础设施体系建设

——加大产业项目投资。坚持以大投资带动大发展，优化投资结构，使工业、基础设施、房地产等投资更趋合理，增强可持续发展能力，避免出现投资泡沫和低效投资。今年拟列入省重点项目37个，年度投资142亿元；安排市重点项目222个（含深汕特别合作区），年度投资415亿元。推动比亚迪零部件、国信通科技、威翔航空、香雪制药、海王药业、陆丰核电、甲湖湾电厂等一批重大产业项目加快建设，力争完成工业投资200亿元以上（不含深汕特别合作区）。加快新一轮企业技术改造，力争完成技改投资95亿元以上，技改项目100个以上。推广PPP模式，鼓励社会资本参与基础设施和社会事业等重点领域，促进民间投资合理增长。发挥财政资金杠杆作用，充分利用创投引导、股权投资等方式，强化政府投资的带动放大效应。

——加快铁公机港等基础设施建设。重点推进广汕高铁、汕汕高铁汕尾段建设，积极开展深汕高铁、龙汕铁路前期工作。加快兴汕高速建设，推动深汕西高速改扩建工程、广东滨海公路汕尾段、珠东快速开工建设。完成国道324陆丰穿城段改建工程，推动国道235陆河河田至河口段、国道324梅陇至鲘门段、省道240陆河至陆丰段、省道510陆丰河西至西南段升级改造。推进小漠国际物流港、陆丰核电一期重件码头建设，优化整合汕尾新港区、海丰港区和陆丰港区。规划海上旅游航线。加快汕尾机场建设前期工作。完成公平灌区节水改造工程，启动公平水库至市区输水工程、龙潭灌区节水改造工程，加快红草园区排洪治涝工程、赤沙水厂等水利基础设施建设。推进城乡电网、甲湖湾电厂出线走廊及一批输变电站等电力基础设施建设。加快新型信息基础建设，推动千兆光纤进小区、百兆光纤进家庭，力争光纤入户率达75%以上，加快有线广播电视网络数字化、双向化、宽带化改造，支持 5G 通信基站建设，加快“智慧城市”进程。

四、加强城市规划建设管理，全力提升中心城区首位度

——以科学规划引领城市发展。启动新一轮城市总体规划修编工作，实现市区近期建设规划范围内控制性详规全覆盖。加快完成市“三旧”改造规划指引编制。在规划编制、审批、实施和监督等环节落实城市设计要求，维护规划的严肃性和权威性。加强土地收储，今年全市计划收储土地5.68万亩，其中市区1.3万亩，为产业园区和中心城区扩容留足空间。

——统筹推进扩容提质。以汕尾新区三个起步区为基本框架，推动中心市区“东拓、西延、北扩、中优”，提升市中心城区首位度和辐射力。东片以中央商务区为中心，向红海湾和赤坑、可塘扩展，升级改造汕遮路，推动红海湾与中心城区一体化发展，做大城市“块头”。西片以“一港一湾”为重点，加快罗马广场至金町湾滨海景观大道、工业大道西段、新衢路建设，升级改造汕马路，推进马宫中心渔港和金町湾滨海旅游度假区建设。北片以红草园区为载体，加快市区与海城双向聚合发展，加快海汕公路升级改造。中部以新中轴线规划建设和环品清湖综合整治开发为抓手，启动新中轴线海绵城市建设，加快碧桂园至罗马广场路段升级改造，实施环湖路绿化景观提升工程，扎实推进环品清湖排污口截污、避风塘清淤疏浚、防波堤改造和非法经营场所清拆，加大力度推进品清湖污染源清理整治，建设最美母亲湖。加快地下综合管廊、市政道路建设，拓宽优化文明路、新华路、翠园街等市政道路，打通金鹏路西段、站前横路西段、育才路等十条断头路，加快青山仔、金台山等城市公园建设，优化提升自来水管网、天然气管道、城市防涝设施等城市配套功能。加快老旧城区改造，推动和顺上村、幸福里等一批城市更新项目建设。建立多主体供应、多渠道保障、租购并举的住房制度，新建一批保障房、人才房，组建市属公租房租赁平台，规范整顿房地产市场秩序，确保房地产市场平稳健康发展，防止大起大落。推进以人为本的新型城镇化建设，加快完善财政、土地、社保、教育等配套政策，积极有序推进非户籍人口在城市落户。

——全面提高城市建设管理品质。举全市之力推进“创文”“创国卫”工作，确保2018年成功创建省文明城市，启动创建国家卫生城市工作。推进城市精细化管理，建设“数字城管”，实施城市“网格化”管理。继续深入推进干线公路、背街小巷、集贸市场卫生环境综合整治，清理整顿占道经营、乱停乱放，全面清理违章户外广告。建立违建巡查管控常态化机制，确保市区违法建筑“零增量”。提升公共交通的多样性和运载能力，完善公共交通运营体系。启动创建国家园林城市，加快城市景观灯光系统建设，提升建设工程标准和质量，加强城市建筑外立面管理，实施城市家具改造，有序建设一批城市雕塑、文化小品，提升城市品位和颜值。

——全力支持深汕特别合作区体制机制调整和开发建设。积极配合做好合作区过渡期管理工作，全力做好体制机制调整后干部群众思想工作和社会维稳工作，尽快做好征地移交，重拳打击违法建设行为。加快推进合作区规划布局调整，推动产城融合发展，重点推进智能制造、机器人、海洋产业等新兴产业发展，加快“四横六纵”主干路网及深东大道、深汕西高速改扩建等对外道路建设，强化水库水厂等城市功能保障。充分发挥深汕特别合作区辐射带动作用，加快深汕一体化发展。

五、深化重点领域改革，加快构建开放型经济新体制

——全面深化改革。扎实推进重要领域和关键环节的改革，推动各项改革任务落地见效。深化“一门式一网式”政务服务模式改革，推广“互联网+商事登记”服务，推进“信用汕尾”平台建设。深化商事制度改革，统筹推进“多证合一”和“证照分离”。设立国有企业改革发展专项资金，打造市级国有资本运营平台，做大做强国有企业。稳妥推进“僵尸企业”重组。继续深化机构和行政管理体制改革、事业单位分类改革。推进环保机构监测监察执法垂直管理改革，成立市、县两级环境保护委员会。启动地方金融监管机构改革，通过压降不良资产、落实捐赠资产、引进战略投资者等综合措施，完成陆河、海丰、陆丰3家农信社改制组建农商行工作。稳妥推进财政事权和支出责任划分改革，全面实施绩效管理。

——加大对外开放力度。主动融入大珠三角经济区，打造粤港澳大湾区辐射延伸区。落实好深莞惠+汕尾河源“3+2”经济圈联席会议重点合作事项。积极复制推广自贸试验区创新措施，推动投资自由化和贸易便利化。培育壮大民营出口企业，大力发展一般贸易，促进加工贸易转型升级。培育外贸新业态新模式，鼓励发展跨境电商、服务贸易和服务外包产业。推进海丰小漠港区、陆丰甲湖港区对外开放，加快埔边快捷通关平台建设，全面推进大通关一体化。

——大力提升企业服务水平。持续推进“放管服”改革，制定出台产业园区投资项目负面清单，建立园区项目多部门联网审批“绿色通道”，提高涉企事项审批服务效率。强化金融服务实体经济能力，发挥中小微企业、优质技改项目等融资风险补偿平台及贷款担保机构作用，推广中小微企业信用信息与融资对接平台应用，解决企业融资难融资贵问题。预判投产企业用工需求，为企业和就业者搭建对接平台，解决企业招工难题。实施中小企业成长计划，大力扶持民营经济发展壮大。激励和保护企业家精神，弘扬工匠精神，支持企业聚焦实体经济加快做大做强。加快市场主体发展，引导“个转企”1500家，培育“四上”企业100家，支持信利光电等有条件的企业上市（挂牌）。

六、实施乡村振兴战略，推进农业农村现代化

——全力推进农业供给侧结构性改革。坚持质量兴农、绿色兴农。不断夯实现代农业基础，推进粮食生产功能区和重要农产品生产保护区划定工作，新建高标准农田3.29万亩，保持粮食储备16万吨，加快海丰、陆丰两个超10万吨优质稻米生产基地建设，保障粮食安全。规划建设提升莲花山茶叶、虎噉金针菜、陆河青梅、华侨红杨桃、陂洋菠萝、凤山红灯笼荔枝等特色农业基地。增加优质绿色农产品供给，扶持培育一批省级名特优新农业特色品牌，争创一批国家级、省级农业标准化示范区，建立市农产品销售平台，开展汕尾名牌农产品评选推介，提升市场竞争力。大力发展农产品精深加工，扶持冷链物流企业发展，拓宽销售渠道，推动农产品加工企业转型升级。发展农业新业态，加快培育旅游观光、农耕体验等休闲农业。大力培育龙头企业、农业专业合作社等新型农业经营主体，发展多种形式适度规模经营，新增市级以上农业龙头企业5家、家庭农场20家。加快农业科技创新，提升科技成果转化率，推进主要农作物生产全程机械化。

——加快发展现代渔业。全力推进马宫中心渔港、遮浪渔港、碣石渔港等现代渔港建设，完成品清湖区域性避风锚地建设。加快汕尾（马宫）海洋渔业科技产业园建设，抓紧建设水产品交易市场。培育海洋新兴产业，支持海洋生物医药研发。深化水产品加工流通，搭建政银担企合作平台，扶持国泰、五丰等龙头企业发展，打响汕尾水产特色品牌。加快捕捞渔船更新改造，组建银鹏、中渔等远洋捕捞船队，发展外深海远洋捕捞作业。发展集约化高效清洁养殖，支持发展深水网箱养殖。挖掘本地特色渔业文化和民俗文化特色资源，创建一批最美渔村和精品休闲渔业示范基地。

——加快新农村建设步伐。加大统筹整合力度，集中力量、集中资源解决乡村振兴突出问题。全力推进142条省定贫困村美丽宜居乡村建设，推动贫困村实现“后队”变“前队”。加快推进农村全域人居环境整治，打造美丽宜居乡村。科学编制村庄发展规划，规范和引导提升村庄建设，严格宅基地管理。抓好农村危破旧房清拆、垃圾污水处理、“四好农村路”建设、“厕所革命”、“空心村”改造等工作，推动农村基础设施建设，确保年底前所有省定贫困村自然村达到创建新农村示范村整治标准。加强古村落、历史建筑、古驿道、红色资源修复保护与开发利用。

——深化农村综合改革。全力推进农村土地确权登记颁证扫尾工作，确保上半年全面完成确权登记颁证任务。完善承包地“三权分置”制度，建立土地流转平台，促进农村土地统一规范有序流转。抓好海丰农村集体产权制度改革试点工作。推进供销合作社综合改革，完成国有林场改革。规范完善农村集体“三资”平台管理，全面实现县、镇、村三级互联互通。推进农村金融和农业保险改革创新。深化小型水利工程管理体制改革。加快推进陆河水唇全国农村综合性改革试点工作。

七、打好污染防治攻坚战，加强生态文明建设

——推进绿色发展。发展壮大节能环保产业、清洁生产产业、清洁能源产业，建立健全绿色低碳循环发展的经济体系。加强对重点耗能工业企业节能管理，确保完成节能降耗任务。推进节约集约用地，加大闲置土地处置力度。倡导简约适度、绿色低碳的生活方式，加大新能源汽车推广力度，推广绿色建筑和建材，大力发展装配式建筑，开展创建节约型机关、绿色家庭、绿色学校、绿色社区和绿色出行等行动。

——加强生态环境治理和保护。坚决打好水、大气、土壤污染防治三大战役，继续做好中央环保督察问题整改落实工作。全力抓好水污染防治，实行最严格水资源管理制度，加快奎山河、奎山湖黑臭水体整治，全面推进市、县、镇、村四级河（库）长制，加快推进45条、290公里中小河流专项整治，启动螺河、黄江和赤石河等重点流域入河排污口整治、河道清淤疏浚和生态修复工程，开展海丰龙津河穿城段、陆丰东河穿城段环境整治，严厉打击非法偷排工业废水行为，加强对公平水库、螺河等集中式饮用水源保护，做好市水资源空间规划，提高水利工程质量。打赢蓝天保卫战，力争空气质量综合指数继续保持全省第一，完成黄标车淘汰任务，严格划定市区高污染燃料禁燃区，加强城市扬尘污染控制，确保建筑工地绿色施工。实施农村环境保护和土壤污染防治行动计划，开展土壤环境详查，有效监控梅陇、可塘的土壤质量，从严控制化肥农药使用，划定畜禽养殖禁养区，依法关闭或搬迁禁养区内的畜禽养殖场。深入实施新一轮绿化大行动，造林30.9万亩、封山育林16.6万亩、森林抚育50万亩，建设乡村绿化美化示范点64个。严守林业生态红线，加快桉树退出。加强山体保护管理和开发利用。严厉打击非法采砂活动。严守海岸带生态保护红线，做好近岸海域污染防治，清理非法设置入海排污口，严格控制、规范管理滩涂，坚决逐步退出近海养殖，规划建设红海湾遮浪国家级海洋公园，加快推动蓝色海湾整治、小岛生态保护修复、龟龄岛开发利用，推进国家级海洋牧场示范区建设。

——补齐环保基础设施短板。启动建设汕尾星都循环经济产业园，争取环保部“无废城市”建设试点落户我市。推进市生活垃圾无害化处理中心二期、陆丰东南生活垃圾焚烧发电厂、市区南区污水处理厂、中心镇污水处理厂、市医疗废物无害化集中处理中心、市建筑垃圾再生利用项目及污泥无害化处理项目建设，两年内完成29个简易镇级垃圾填埋场整改工作，提升垃圾、污水等综合处理能力。完善污水处理设施配套管网，推动雨污分流，新增管网300公里，确保污水处理厂运营负荷率达75%以上。新建、改造一批公共厕所、旅游厕所和窗口单位厕所，实现旅游厕所全域覆盖。

八、全面发展各项社会事业，加快补齐民生短板

——增强就业和社会保障能力。按照兜底线、织密网、建机制的要求，不断增加就业岗位，实现城镇新增就业5万人，新增转移农村劳动力就业2.8万人，城镇登记失业率控制在3%以内。抓好企业职工基本养老保险扩面征缴工作，稳妥推进机关事业单位养老保险制度改革，逐步提高社会保险待遇和均衡水平。发挥医保撬动作用，推动医疗、医药、医保“三医联动”改革。稳妥推进医保城乡一体化改革，优化大病保险制度，把困难群体报销比例提高到75%以上。完善社会救助体系，抓好陆河特困人员供养服务机构社会化改革试点工作。

——加快教育现代化步伐。优先发展教育事业，增加各级政府教育经费预算。全力推进教育“创现”工作，确保各县（市、区）上半年申报验收。大力发展普惠性幼儿园，新增幼儿园29所，全面清理整治无证幼儿园。大力扶持特殊教育、民办教育，推行集团化办学模式，完善学生资助体系，强化德育教育工作，建立健全义务教育质量评价体系，努力让每个孩子都能享有公平而有质量的教育。实施高中教学质量提升工程，力争高考成绩实现新突破，本科上线人数突破8000人，本科率达35%，推进市林伟华中学、新城中学创建成为国家级示范性普通高中，加快彭湃中学、龙山中学、河口中学、白沙中学、华侨中学等学校升级提质。新建改建一批职业学校，推进陆河县职业技术学校创建省重点中职学校，探索建立学生分流接受职业技能教育机制，深化产教融合、校企合作，推行“双元制”职业教育模式，大幅提升职业教育占高中阶段教育的比重。加快市高级技工学校和市职院新校区建设，推动工科应用型大学加快落地，办好市开放大学。突出抓好队伍建设，推行基础教育“县管校聘”制度，继续大力整顿师德师风，实行教师退出机制和校长末位淘汰机制。

——加快发展医疗卫生事业。加快卫生强市创建工作，提高公共卫生服务水平。深化医疗卫生体制改革，建立优质高效医疗卫生服务体系，全面推进医联体建设，推动优质医疗服务资源下沉。高质量建设中山大学深汕中心医院，全力打造粤东医疗高地。加快甲子、碣石和梅陇3家综合性医院建设，推动10家县级人民医院升级改造。加快市、县两级中医医院建设，新建市妇幼保健院，支持民营医院发展，规范引导社会办医。推广“互联网+医疗”新模式，打造县级医院与市外三级甲等医院远程医疗平台。全面对接省、深圳医院对口支援，引进医学优势学科和优秀人才，加强重点专科建设和全科医生培养。优化医疗卫生资源配置，推行分级诊疗和基层首诊，力争县域内住院率达到90%以上，基本实现小病不出镇、大病不出县，实现每千常住人口执业（助理）医师数、注册护士数、床位数和每万人全科医生数分别达到1.75名、1.6名、3.05张和2.15名。树立“大健康”理念，大力开展爱国卫生运动，做好重大疫病防控，开展出生缺陷综合防控、免费孕前优生健康检查，免费为7.6万名农村妇女实施“两癌”检查。

——全力打好精准脱贫攻坚战。围绕“脱真贫、真脱贫”目标，按照“聚焦再聚焦、精准再精准”要求，落实好人盯人、人盯户帮扶联系机制，确保帮扶更加精准有效。把扶贫和扶志、扶智结合起来，打好产业、就业、物业帮扶及民生兜底政策组合拳，激发贫困人口内生动力，推进精准扶贫、精准脱贫各项政策措施落地生根，确保3.7万相对贫困人口全面脱贫。加强动态管理，对新致贫和返贫人口及时纳入帮扶。按“一镇一业”“一村一品”要求，因地制宜引导贫困村发展特色产业，加大农业旅游、金融、光伏、电商等扶贫开发力度，带动贫困户增收脱贫。加强贫困人口就业培训，鼓励各类企业吸纳贫困劳动力，公益性岗位优先安排贫困人口。落实贫困人口教育、医疗、住房“三保障”政策，推动兜底保障政策落地见效。

——推动文体及其他事业发展。加强精神文明建设，坚持用社会主义核心价值观凝聚共识、汇聚力量。擦亮汕尾“中国民间艺术文化之乡”品牌，宣传推广汕尾文化。加强文物和文化遗产保护传承，做大做强文化产业，提升文化软实力。精心筹办建市三十周年系列活动、第三届广东（汕尾）“马思聪杯”小提琴邀请赛、市第七届文化惠民活动周等大型文化活动。挖掘海陆丰红色文化资源，推进红宫红场、彭湃故居等一批革命遗址的保护开发利用，争取省的支持，建设省级干部学院，打造更多革命传统教育基地。广泛开展群众性体育赛事活动，举办市首届马拉松比赛，全力备战第十五届省运会，引进皮划艇、龙舟、帆船帆板等传统优势项目高水平人才，扶持一批特色体育项目，提高竞技体育水平。全面建成市体育中心，加快省帆船帆板（红海湾）基地升级改造，推进市民文化中心、市工人文化宫建设，加快市体校和业余体校新校区建设。动工建设市福利中心，加快镇级公益性公墓山（骨灰楼）建设，确保陆河县殡仪馆如期完工。

同时，注重解决民生突出问题，办好十件民生实事。

九、加强社会治理体系建设，维护平安和谐稳定大局

——不断提升社会治安防控能力。按照省委十二届二次、三次全会提出的“广东任何一块土地上都必须是清明世界、朗朗乾坤，决不许藏污纳垢，要让恶势力无任何立锥之地，望而却步，闻风而逃，让各类犯罪分子无所遁形”的要求，构建“大侦查、大防控、大服务、大保障、大监督”工作格局，坚持重拳惩恶，严厉打击黄赌毒黑拐骗和制假售假等各类突出违法犯罪活动，建设法治汕尾、平安汕尾。保持禁毒力度不减，建立健全长效机制，严防毒情反弹回潮。建立常态化管控机制，防止碣石“洋垃圾”问题死灰复燃。构建立体化、信息化社会治安防控体系，加快“雪亮工程”建设，有效推进陆丰、海丰社会治安重点地区整治。推进信访工作法治化建设，坚持依法彻底解决乌坎涉稳、土地争议等问题。加强对涉军、涉农、涉土、涉环保、涉劳资纠纷等不稳定因素排查化解，确保不发生重大群体性事件。加强城市安全和公共安全工作，健全应急管理体制，提高突发事件预防预警和应急处置能力。加强防灾减灾救灾体系建设，全面完成三防系统标准化建设，推进“五个一”救灾平台建设。深刻汲取公平“12·9”火灾事故血的教训，增强安全生产红线意识和风险管控意识，落实企业安全生产主体责任和行业监管责任，以更大力度消除各类安全隐患、堵塞各类安全漏洞。加强食品药品、重点工业产品、危爆物品、特种设备等安全监管，继续加大打击假冒伪劣产品力度。打好防范化解重大风险攻坚战，开展护航金融“利剑行动”，坚决打击非法集资活动，严守不发生系统性区域性金融风险底线。做好国防动员、人民防空、双拥共建、优抚安置等工作。

——有效加强基层治理。大力培育自然村（村民小组）村民理事会，加强村（居）务公开“五化”和村务监督委员会“九有”建设，建立健全村（居）民民主议事制度，发挥村（社区）公共服务中心（站）作用，增强村“两委”干部素质和本领，全面提升基层自治能力和服务水平。完善一村（社区）一法律顾问制度。培育规范各类社会组织，培育孵化一批青年组织，壮大志愿者等服务队伍力量。

各位代表！全面完成今年各项目标任务，必须坚持和加强党的全面领导，切实增强“四个意识”，牢固树立“四个自信”，更加自觉在思想上政治上行动上同以习近平同志为核心的党中央保持高度一致，严守政治纪律和政治规矩，全面彻底肃清李嘉、万庆良流毒影响，全面推进政治建设、思想建设、组织建设、作风建设、纪律建设，把制度建设贯穿其中，加快政府职能转变，建设人民满意的服务型政府。一是建设法治政府。自觉接受市人大及其常委会工作监督和法律监督，积极支持人民政协履行政治协商、民主监督、参政议政职能。高质量办理人大代表建议和政协委员提案。大力发展协商民主，认真听取各民主党派、工商联、无党派人士和各人民团体的意见建议。主动接受群众、媒体和审计监督。扎实推进“七五”普法，推进各级领导干部学法常态化，做好政务公开，做好地方性法规草案审修和政府规章制定，推动行政机关负责人出庭应诉工作，高标准建设法治政府。二是建设高效政府。对标广州、深圳等珠三角地区，全面优化政务服务，精简行政审批事项，推动流程再造。大力推进“数字政府”建设，让数据多跑路、群众少跑腿。弘扬“马上就办、真抓实干，办就办好、滴水穿石”的实干精神，加强部门协作，落实重大项目和重点工作限时办结制度，完善绩效考核和督查问责机制，以问题为导向倒逼工作落实，建设高效型行政机关。三是建设廉洁政府。推进“两学一做”学习教育常态化制度化，扎实开展“不忘初心、牢记使命”主题教育。严格落实中央八项规定及修订后的实施细则、省委实施办法和市委实施意见，驰而不息纠正“四风”，严格控制“三公”经费，下大力气整治文山会海。深入推进政务整治、正风肃纪行动，重点整治“中梗阻”“为官不为”“庸懒散奢”“吃拿卡要拖”等突出问题，加强重点领域和关键岗位廉政风险防控工作。建立健全激励机制和容错纠错机制，推进能者上、庸者下、劣者汰，打造一支忠诚干净、担当作为、勤政廉洁的干部队伍。

各位代表！新时代开启新征程，新时代呼唤新作为。让我们更加紧密地团结在以习近平同志为核心的党中央周围，高举习近平新时代中国特色社会主义思想伟大旗帜，认真贯彻落实党的十九大精神和习近平总书记对广东工作的重要指示批示精神，按照省委、省政府和市委的决策部署，不忘初心、牢记使命，以永不懈怠的精神状态和一往无前的奋斗姿态，把汕尾建设成为宜居宜业宜游现代化滨海城市，为全面建成小康社会、开启全面建设社会主义现代化新征程而努力奋斗！
